# Preparation and *In Vitro* Characterization of Enoxaparin Nano-liposomes through Different Methods

**DOI:** 10.34172/apb.2021.042

**Published:** 2020-08-05

**Authors:** Sarveen Palassi, Hadi Valizadeh, Saeideh Allahyari, Parvin Zakeri-Milani

**Affiliations:** ^1^Biotechnology Research Center and Faculty of Pharmacy, Tabriz University of Medical Sciences, Tabriz, Iran.; ^2^Drug Applied Research Center and Faculty of Pharmacy, Tabriz University of Medical Sciences, Tabriz, Iran.; ^3^Student Research Committee, Faculty of Pharmacy, Tabriz University of Medical Sciences, Tabriz, Iran.; ^4^Liver and gastrointestinal Diseases Research Center and Faculty of Pharmacy, Tabriz University of Medical Sciences, Tabriz, Iran.

**Keywords:** Enoxaparin, Nano-liposome, Methods, Characterization, Azure II

## Abstract

***Purpose:*** Enoxaparin has been widely used as a choice drug for treatment and prevention of different coagulation disorders. Orally administered enoxaparin encounters with gastrointestinal barrier because of its high water solubility, high molecular weight and significant negative charge. Since, the nano-liposomes has gained great interest for oral drug delivery, we decided to introduce the best protocol for preparing enoxaparin nano-liposomes through *in vitro* characterization.

***Methods:*** Nano-liposomes were prepared by ethanol injection, thin film hydration, and double emulsion/solvent evaporation methods. Size distribution, zeta potential, loading efficiencies, and *in vitro* drug release of nano-liposomes were also studied.

***Results:*** The mean vesicle size was obtained under 100 nm, and the zeta potential was highly negative through all preparation methods. Nano-liposomes prepared by double emulsion/ solvent evaporation (DE) technique could entrap more of this hydrophilic drug (43 ± 7.1 %), but through thin layer hydration (TL) and ethanol injection (EI) only 28.4± 3.2% and 17.3 ± 2.5% of drug could be loaded into synthesized carriers. Drug release from these carriers was also obtained 42.17±1.72%, 29.43±0.34% and 32.27±0.14%, in 24 hours for EI, TL, and DE methods, respectively.

***Conclusion:*** Here, we can introduce double emulsion/solvent evaporation method as an acceptable method for enoxaparin loading, although some toxicity and in-vivo tests are also necessary to fully understand the potential of this formulation.

## Introduction

Enoxaparin as a low molecular weight heparin (4.5 kDa) plays an important therapeutic role in deep-vein thrombosis, pulmonary embolism, unstable angina, and dialysis.^1^ It can also be prescribed for treatment and prevention of deep vein thrombosis and pulmonary embolism during pregnancy in a safe manner instead of warfarin which causes fetal warfarin syndrome.^2^

Enoxaparin has the ability of anticoagulation because of its highly sulfated pentasaccharide part which binds to antithrombin and catalyzes the inactivation of factor Xa.^3^ This anionic (due to sulfate and carboxylate groups) high molecular weight drug has high water solubility (200 mg/mL) and low absorption through gastrointestinal membrane, so it loses its activity in acidic medium of stomach when is orally administered.^4^ Therefore, it is used through intravenous or subcutaneous pathway which may cause patient discomfort, pain, hematoma, and infection. Because of its short half-life, injection might have to be repeated in a day which involves more restrictions and high costs for the patients.^5^

Development of nano-liposome for oral drug delivery has gained great interest in pharmacy. This sphere-shaped vesicle with at least one lipid bilayer, has an aqueous core surrounded by a hydrophobic membrane is used as a perfect model of cells and bio membranes.^6^ Enoxaparin with its hydrophilic part can be incorporated in the aqueous core of the liposome. This carrier not only has the ability to enhance bioavailability and stability of oral drugs, but also minimize their unwanted interactions.^7^

Up to this date, topical delivery of flexible nano-liposomal enoxaparin,^8^ inhalable enoxaparin liposomes for the treatment of pulmonary embolism,^9^ and mucoadhesive nanoparticles of enoxaparin for skin ulcers have been studied.^10^ Also, nano-liposomes of Enoxaparin were prepared based on enzyme-substrate interaction for lymph node metastasis but in parenteral route of delivery.^11^ In present study nano-liposomal formulation of Enoxaparin was prepared through various pathways to compare their effects on different in vitro characteristics of drug and liposome.

## Materials and Methods

### Materials

Egg lecithin, cholesterol (as nano-liposomal ingredients) and enoxaparin sodium were obtained from Merck company (Darmstadt, Germany). Azure II was supplied by Elder Pharmaceutical Ltd. (Mumbai, India). Purified water was obtained from MilliQ Plus (Millipore Corporation, Billerica, MA). Ethanol and chloroform had been used as solvents which were purchased from Merck Company (Darmstadt, Germany). Disodium phosphate and sodium dihydrogen phosphate, used for preparing phosphate buffer and were also purchased from Merck Company (Darmstadt, Germany). The materials were used without further purification.

### Methods

All measurements were done in triplicate and errors were shown by standard deviation.

### Preparation of nano-liposomes

Nano-liposomes were prepared by three methods including ethanol injection,^12^ thin film hydration,^13^ and double emulsion/solvent evaporation technique.^14^ The preparation methods are explained exactly in following sections. We also define all ratio of materials that were used during formulations in Table 1.

**Table 1:** Demonstrating all ratio of materials used for enoxaparin- nanoliposome formulations through ethanol injection (EI), thin layer hydration (TL), and double emulsion/solvent evaporation (DE) processes

	**EI**	**TL**	**DE**
Lecithin	160 mg (2×10^-4^ mol)	160 mg (2×10^-4^ mol)	160 mg (2×10^-4^ mol)
Cholesterol	40 mg (1×10^-4^ mol)	40 mg (1×10^-4^ mol)	40 mg (1×10^-4^ mol)
Lecithin/cholesterol	2:1	2:1	2:1
Enoxaparin	20 mg	20 mg	20 mg
Distilled water	20 mL	20 mL	1 mL
Phosphate buffer	-	-	19 mL
Solvent	2 mL ethanol	5 mL ethanol	5 ml chloroform

**Figure 1:**
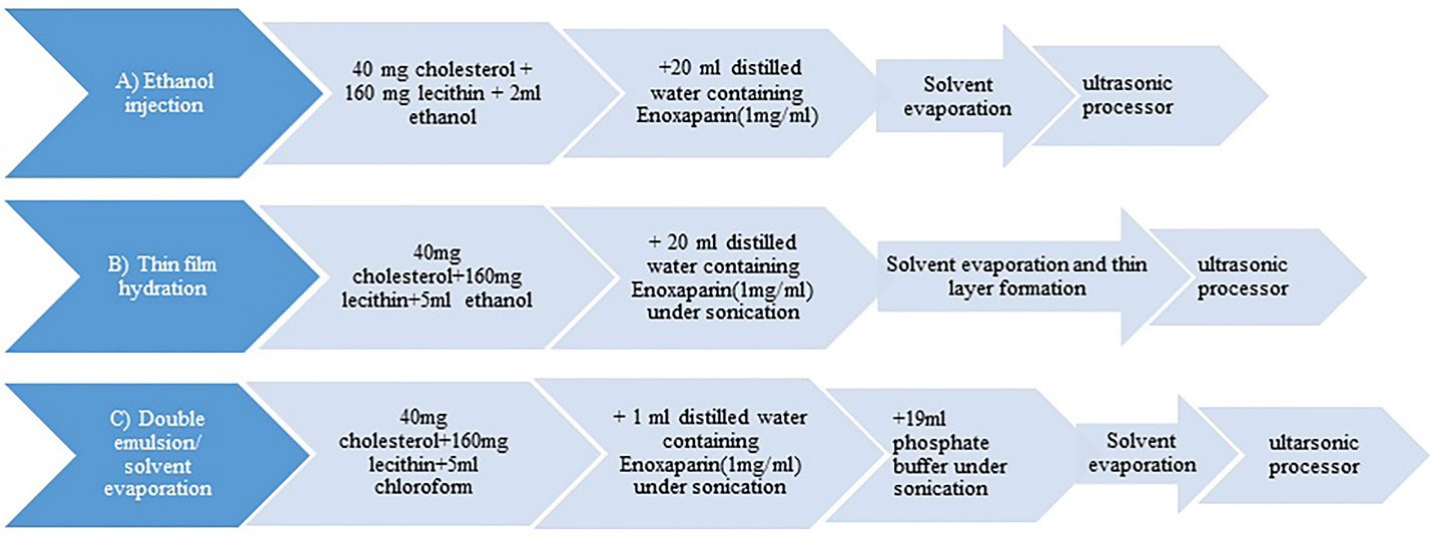
Flow chart demonstration of preparation steps. All steps are done at 60±2°C.

Ethanol injection method: In this method specific ratio of egg lecithin and cholesterol were dissolved in 2 mL ethanol at 60°C while stirring under heater stirrer (Thermo Mantle, Amsterdam, Holland) in order to obtain the organic phase. 20 mg of enoxaparin was dissolved in 20 mL of preheated purified water (aqueous phase). Then, the organic phase was injected by a syringe rapidly into the aqueous phase under stirring at 1200 rpm.^12^ After injection, the sample remained stirring for 30 minutes and then transferred to the rotary evaporator (Heidolph, Schwabach, Germany) for 2 hours (160 mm Hg), at 60°C, to remove part of ethanol. The obtained sample was applied to the probing sonication (CT-chromtech, Taipei, Taiwan) for 30 minutes (6 seconds on, 3 seconds off). Then it was filtered through 0.22 µm Sartorius filter. To make sure that all of the ethanol was evaporated the sample was dialyzed using dialysis bag (MW cut-off 12-14KD) for 24 hours, twice at +4°C^15^ (Figure 1A).

Thin film hydration method: Thin film layer was formed by rotary evaporator at 210 mm Hg and 60°C from organic phase which was prepared as described above with specific amounts of cholesterol and lecithin. The dried film layer was hydrated with 20 mL water containing the drug (1 mg/mL) under sonication.^16^ To reduce the particle size, the latter sample remained under sonication for 30 minutes. Then it was taken to ultrasonic processor like the previous method. Finally, it was filtered and stored at +4°C^17^ (Figure 1B).

Double emulsion/solvent evaporation method: Briefly, cholesterol and egg lecithin were dissolved in 5 mL chloroform and 20 mg of the drug was prepared in 1ml water at 60°C. Then aqueous phase was transferred in to the organic medium under sonication (Digitec, Hamburg, Germany) forming the primary emulsion. This emulsion was transferred into 19 mL of phosphate buffer in order to form the double emulsion water-in-oil-in-water (W_1_/O/W_2_). The organic solvent was totally evaporated (170 mm Hg) by rotary evaporator.^10^ Then the sample was taken to ultrasonic processor as described earlier. All steps were done at 60°C. Finally, it was filtered and kept at +4°C (Figure 1C).

### Size distribution and zeta potential

Size distribution and zeta potential of the nano-liposomes were characterized by dynamic light scattering (Malvern Instruments, Worcestershire, UK) without any dilution.^18^

### Entrapment efficiencies

In order to separate unloaded enoxaparin, ultracentrifugation (Optima TLX, Beckman, Calif, USA) technique with 139104 g was done at the room temperature (25±2°C). Then the precipitant was collected and kept in phosphate buffer. A part of the sample was used to determine loading parameters in direct method. To destruct the structure of nano-liposomes the same volume of chloroform was added to the suspension and shacked well to form two phases. Then, 500 µL of the supernatant was added to 4.5 mL Azure II solution (0.01mg/mL).^19^ Enoxaparin concentration was assessed through a calibration curve obtained from UV/visible spectrophotometer (UV 1800, Shimadzu, Kyoto, Japan) at 513 nm. The entrapment efficacy was calculated using the following equation:

$$\%\;Encapsulation\;efficiency=\frac{Total\;amount\;of\;entrappe\;drug\;(g)}{Actual\;amount\;of\;added\;drug\;(g)}\times 100$$

### In-vitro drug release

This test was done by dialysis method (MW cut- off 12-14 KDa) during 24 hours. 2 mL of prepared sample was transferred to a micro dialyzer in 78 mL of phosphate buffer (pH= 6.8) as receiving compartment under heater stirrer at 37°C. 500 µL of the release medium was withdrawn every 30 minutes and replenished with the same volume of fresh buffer. The samples were added to 4.5 mL Azure II and vortexed to be analyzed by spectrophotometer at 513 nm.^18^

The obtained drug release data were analyzed mathematically with zero-order, first-order, Higuchi, Hixon-Crowell, and Korsmeyer–Peppas equations:^20^

Qt=k0t+Q0lnQt=lnQ0−k1tQt=kHt12Q013−Q13=kHCtQtQ∞=kKPtn

Where Q_t_ is the amount of drug released at time t; Q_0_ is the initial amount of the drug in the formulation; Q_∞_ is the amount released at the equilibrium state; k_0_, k_1_, k_H_, and k_HC_ are release rate constants for zero-order, first-order, Higuchi, and Hixson-Crowell rate equations.; k_KP_ is the kinetic constant, and n is the exponent of release.

### Dissolution parameters

Dissolution parameters including DE (dissolution efficiency), MDR (mean dissolution rate), difference (f_1_) and similarity (f_2_) factors were calculated by the following equations as shown in Table 2.

**Table 2:** Dissolution parameters equations (DE (dissolution efficiency), MDR (mean dissolution rate), difference factor (f1), and similarity factor (f2)).

**Dissolution parameters**	**Equation**	**Definition**
DE	$DE=\frac{\int_{0}^{t}y_{t}\;.dt}{y_{100}\;.t}\times 100$	Area under the dissolution curve up to the time, t. Represented as the percentage of the area of the rectangle where y is the percent of drug dissolved at time t.
MDR	$MDR=\frac{\sum_{j=1}^{n}\frac{\Delta M_{j}}{\Delta t}}{n}$	n is the number of dissolution sample times, Δt is the time at midpoint between t and t−1 (easily calculated with (t + t−1)/2) and ΔM_j_ is the additional amount of drug dissolved between t_j_ and t−1.
f_1_	$f_{1}=\frac{\sum_{t=1}^{n}\left|R_{t}-T_{t}\right|}{\sum R_{t}}\times$	n is the number of time points, R_t_ is the dissolution value of the reference formulation at time t and T_t_ is the dissolution value of the test formulation at time t. f_1_, is a measurement of difference in percent (%) between the dissolution curves.
f_2_	$f_{2}=50\;\log\;\left\{{\left[1+\frac{1}{n}\;\sum_{t=1}^{n}{\left(R_{t}-T_{t}\right)}^{2}\right]}^{-0.5}\times 100\right\}$	n is the number of time points, R_t_ is the dissolution value of the reference formulation at time t and Tt is the dissolution value of the test formulation at time t. f_2_, is a logarithmic reciprocal square root transformation of the sum of squared error and is a measurement of the similarity in percent (%) between the dissolution curves.

## Results and Discussion

### Size distribution and zeta potential

Particle size has been known as an important factor for gastrointestinal absorption through endocytosis and paracellular pathways. Size distribution by volume, PDI (polydispersity index) values and zeta potentials of the loaded nano-liposomes are shown in Table 3.

**Table 3:** Particle size and loading characteristics of enoxaparin loaded nano-liposomes

**Method**	**d (nm)**	**PDI**	**z (mV)**	**LE (% w/w)**
EI	49.42 ± 18.97	0.282 ± 0.011	-30.7 ±7.25	17.3 ± 2.5
TL	63.61 ± 13.11	0.295± 0.009	-26.7 ± 5.06	28.4 ± 3.2
DE	46.34 ± 20.2	0.456± 0.006	-27.9 ± 7.41	43 ± 7.1

***Note***. Mean values ± standard deviation, n=3. EI, ethanol injection; TL, thin layer hydration; DE, Double emulsion/solvent evaporation; d, mean diameter by volume, z, zeta potential, PDI, polydispersity index; LE, loading efficiency.

It can be understood that the mean diameter for nano-liposomes was obtained under 100 nm for all three methods. This size range can create high surface area for absorption. PDI results as a homogeneity factor, showed better results in ethanol injection method. Maitani et al also represented that by using extrusion method this parameter would be improved.^21^ When ethanol is added to the aqueous phase through a needle, it becomes diluted and forces the dissolved lipids to self-assemble spontaneously and forms small unilamellar vesicles.^22^ But in the other two methods MLVs (multilamellar vesicles) are created firstly and we have to add aqueous phase to organic phase under bath sonication in order to form small unilamellar vesicles.

Zeta potential results indicated that electrostatic forces between the sample particles have optimum stability in the suspensions and sufficient stability would be gotten in higher negative values of 20 mV.^23,24^ The zeta potential value of nano-liposomes was related to the interaction between the hydroxyl groups of polyanion enoxaparin and choline part in the polar area of phosphatidylcholine which creates the dipole tropism and rises the particle surface charges.

### Entrapment efficiencies

Drug entrapment efficiencies are also shown in Table 3. Loading efficiency is known as a key role in liposomal formulations. Poor entrapment of highly water soluble drugs is known as a major barrier in preparation of loaded nano-liposomes. Double emulsion/solvent evaporation method, resulted in higher loading efficacy for this kind of drugs such as enoxaparin (Table 3). Previous studies also indicated this method had advantages in encapsulating of hydrophilic drugs with high entrapment efficiencies.^25^ It is maybe because of liposomes creation during chloroform evaporation. When chloroform is evaporated two layers of the membrane gets thinner and makes more space for hydrophilic drug to be entrapped inside.^26^ Aqueous phase volume, membrane rigidity, particle size and preparation methods are important factors that may affect the encapsulation efficiency. A possible weakness of double emulsion/solvent evaporation method is the existence of residual chloroform in the final product which is toxic and may also affect the stability of liposomes.^27^ This problem can also occur in ethanol injection method. Ethanol injection method resulted in the lowest entrapment. This method forms smaller particles even before using ultrasonic processor when compared with other methods. Therefore, it has less space to encapsulate the hydrophilic drug.^28^

Nee Ling et al revealed that cefotaxime as a highly water soluble drug could be entrapped efficiently in pro nano-liposomes and resulted in 2.7 times greater bioavailability in comparison with that of the aqueous solution. Lymphatic transport of the drug was also promoted by creating liposomal form of the drug and 23.9 ± 1.0% of the drug was entrapped in the mentioned carriers.^29^

### In vitro drug release study

**Figure 2:**
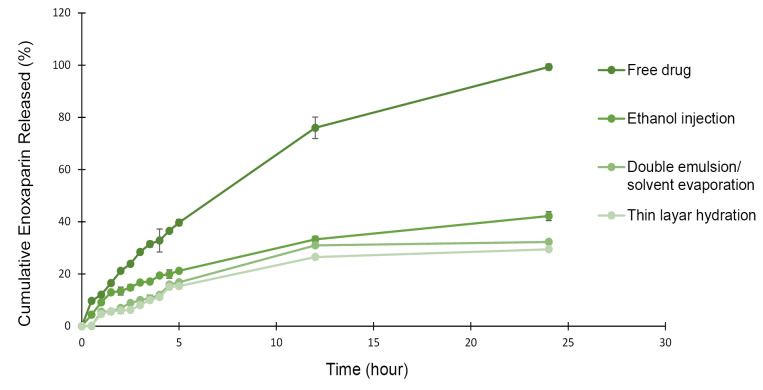
Release profiles of enoxaparin loaded nano-liposomes in phosphate buffer solution (pH=6.8) at 37°C ±2°C.

The cumulative drug release profiles data is shown in Figure 2. It would be desirable if the release procedure starts only when the nano-liposomes reach and interact with the intestinal tissue. The *in vitro* drug release is not reliable in acidic medium due to Enoxaparin’s instability in acidic pH^19^ so we studied in phosphate buffer solution (pH=6.8) at 37°C. The release profile of free enoxaparin shows 100% diffusion within 24 hours. Entrapment phenomenon may reduce not only the cumulative release, but also the release rate of loaded enoxaparin. Nano-liposomes made by ethanol injection, thin film hydration, and double emulsion/solvent evaporation methods could release 42.17±1.72%, 29.43±0.34%, and 32.27±0.14% in 24 hours, respectively. Less drug release for thin hydration method is maybe due to nonexistence of organic solvent. Presence of ethanol in Ethanol injection method can make more space between phospholipid molecules and lead to more permeable liposomes.^30^ Hales et al indicated that enoxaparin sodium loaded polymeric microspheres prepared by double emulsion/solvent evaporation technique released 15.16% of the drug in 5 hours.^4^ Another study reported that liposome formulation of 5-fluorouracil showed almost 70% release in isotonic phosphate buffer saline (pH=7.4) medium. This hydrophilic class III drug was prepared by thin film hydration method.^31^ Metronidazole as a BCS class I drug was also demonstrated 7.24% release from intact proliposomes in phosphate buffer solution (pH=4.5).^32^ These results are indicated that method of preparation, type of drug, and medium properties are important factors that may influence on extent and profile of drug release.

The release profile was also characterized mathematically using zero-order, first-order, Higuchi, Hixon-Crowell, and Korsmeyer–Peppas equations to define the release kinetics of formulations.

Based on computed correlation coefficients, Enoxaparin release from liposomes which were prepared through DE and TL processes, followed first-order kinetic (R^2^ 0.92 and 0.87, respectively), indicating that the change in concentration with respect to change on time was dependent only on concentration. However, the EI method followed Higuchi release phenomenon (R^2^ 0.97), indicating a diffusion controlled release of drug.

### Dissolution parameters

Independent dissolution factors including DE and MDR usually show dissolution rate of a samples throughout the dissolution procedure.^33^ The calculated dissolution parameters are shown in Table 4.

**Table 4:** Dissolution parameters ethanol injection (Ⅰ), double emulsion/solvent evaporation (ⅠI), thin layer hydration (IIⅠ)

**Method**	**DE (%)**	**MDR (mg/min)**
Ⅰ	29.68	0.057
Ⅱ	24.52	0.046
Ⅲ	21.62	0.042
**Compared methods**	**f**_1_ ** (%)**	**f**_2_ ** (%)**
Ⅰ-Ⅱ	44.28	61.38
Ⅰ-Ⅲ	62.37	56.85
Ⅱ-Ⅲ	11.42	83.82

*Note*. DE, dissolution efficiency; MDR, mean dissolution rate; f_1_ and f_2_, difference and similarity factors.

DE and MDR for the Ethanol injection method were achieved 29.68% and 0.057 (mg/mL), respectively. That showed fast drug release from nano-liposomes in comparison with other two methods.

Fit factors (f_1_ and f_2_) were also calculated to demonstrate differences between corresponding values in the two curves. When f_1_ values are less than 15% and f_2_ values are greater than 50%, it means that those curves are statically considered similar.^34^ As shown in Table 4, curves representing double emulsion/solvent evaporation and thin layer hydration methods, are almost similar.

## Conclusion

Encapsulation of Enoxaparin in nano-liposomes, can give a chance to enoxaparin to be administered orally. Nano-liposomes made by double emulsion/solvent evaporation method are ideal carriers for delivery of hydrophilic molecules which could result in nanosized liposomes with suitable loading efficiencies and drug release profiles.

## Ethical Issues

Not applicable.

## Conflict of Interest

There is not reported any conflict of interest by the authors.

## Acknowledgments

The National Intitue for Medical Research (NIMAD, Grant No. 963499) funded this article.
